# On-Chip Reconfigurable and Ultracompact Silicon Waveguide Mode Converters Based on Nonvolatile Optical Phase Change Materials

**DOI:** 10.3390/nano12234225

**Published:** 2022-11-28

**Authors:** Yedeng Fei, Yin Xu, Dongmei Huang, Yue Dong, Bo Zhang, Yi Ni, P. K. A. Wai

**Affiliations:** 1 Department of Electronic Engineering, School of IoT Engineering, Jiangnan University, Wuxi 214122, China; 2 Institute of Advanced Technology, Jiangnan University, Wuxi 214122, China; 3 The Hong Kong Polytechnic University Shenzhen Research Institute, Shenzhen 518057, China; 4 Photonics Research Institute, Department of Electrical Engineering, The Hong Kong Polytechnic University, Hong Kong, China; 5 Department of Physics, Hong Kong Baptist University, Hong Kong, China

**Keywords:** silicon photonics, integrated optical devices, higher-order modes, reconfigurable mode converters, phase change materials

## Abstract

Reconfigurable mode converters are essential components in efficient higher-order mode sources for on-chip multimode applications. We propose an on-chip reconfigurable silicon waveguide mode conversion scheme based on the nonvolatile and low-loss optical phase change material antimony triselenide (Sb_2_Se_3_). The key mode conversion region is formed by embedding a tapered Sb_2_Se_3_ layer into the silicon waveguide along the propagation direction and further cladding with graphene and aluminum oxide layers as the microheater. The proposed device can achieve the TE_0_-to-TE_1_ mode conversion and reconfigurable conversion (no mode conversion) depending on the phase state of embedded Sb_2_Se_3_ layer, whereas such function could not be realized according to previous reports. The proposed device length is only 2.3 μm with conversion efficiency (CE) = 97.5%, insertion loss (IL) = 0.2 dB, and mode crosstalk (CT) = −20.5 dB. Furthermore, the proposed device scheme can be extended to achieve other reconfigurable higher-order mode conversions. We believe the proposed reconfigurable mode conversion scheme and related devices could serve as the fundamental building blocks to provide higher-order mode sources for on-chip multimode photonics.

## 1. Introduction

Silicon-on-insulator (SOI), a vital and mature material platform for silicon photonics, has pushed the development of photonic integrated circuits (PICs) based on its high refractive index contrast and CMOS compatible processing [1,2]. On-chip optical interconnects, data centers, and optical communications have been benefited greatly from the compact size, higher performance, and lower power-consumption of PICs [3,4,5]. Most of the current PICs operate in the single-mode state to avoid mode crosstalk and simplify the device design. However, the intrinsic mode degree of freedom of light is lost because of the single-mode operation. To satisfy the rapidly increasing demand on capacity, various on-chip multiplexing technologies have been developed, such as wavelength-division-multiplexing (WDM) [6,7], polarization-division-multiplexing (PDM) [8,9], and mode-division-multiplexing (MDM) [10,11], where the underlying mechanisms are based on the intrinsic properties of light (wavelength, polarization, and mode, respectively). Among these multiplexing technologies, WDM requires expensive multi-wavelength lasers and PDM has only two polarization states to be multiplexed [10,11,12]. In comparison, MDM is best suited for the on-chip multiplexing transmission since higher-order modes could provide new multiplexing channels for the on-chip MDM transmission and reveal unique features compared with commonly used fundamental modes [12]. Multimode photonics is an emerging research field in silicon photonics, and higher-order mode generators or converters represent one of the fundamental components to generate the higher-order mode sources for on-chip multimode applications [13,14].

Recently, various silicon waveguide mode converters based on different device structures or adding extra materials have been reported. For mode converters, Mach-Zehnder interferometer (MZI) waveguide and asymmetrical directional coupler (ADC) are the commonly used configurations [15,16]. MZI type mode converters normally require mode splitting, phase difference accumulation, and recombination, thus resulting in relatively long device lengths (e.g., >50 μm [15]). ADC type mode converters require the input mode and desired output mode to satisfy the wavelength-dependent phase matching condition, resulting in narrow working bandwidths and tight fabrication tolerances [16]. Mode conversion on a single strip waveguide avoids these drawbacks. For example, by introducing deeply or shallowly etched slots on the silicon waveguide, conversion from fundamental TE_0_ (TM_0_) mode to higher-order TE_1_ or TE_2_ (TM_1_ or TM_2_) modes have been demonstrated. The mode conversion length can be reduced to less than 10 μm [17,18,19,20]. Methods, such as inverse design [21], deep learning [22], and topology optimization [23], have been employed to find the optimum structure for mode conversion based on the silicon waveguide. However, the time-consuming iterative calculations and the device structure generated might pose a challenge for device fabrication [21,22,23]. For the mode conversion material, it has been shown that metal plasmonic materials are able to clearly change the mode distributions in the silicon waveguide and achieve the designed mode conversions [24]. High absorption loss, however, is the main disadvantage of these mode converters even for hybrid plasmonic waveguide structures [24,25]. Other high refractive index materials have also been embedded in the silicon waveguide to achieve mode conversion, but high transmission loss and complex fabrication processes remain the obstacles to be overcome [26]. Most of the reported mode converters to date perform a single mode conversion function only. They cannot perform two or more mode conversion functions, and are not reconfigurable, extensible, or programmable either [15,16,17,18,19,20,21,24,25,26]. These functions however are essential for the realization of on-chip multimode photonics.

Phase change material (PCM), such as widely used Ge_2_Sb_2_Te_5_ and Ge_2_Sb_2_Se_4_Te_1_, has tremendously different optical properties between the amorphous and crystalline phases. Both phase states can be transformed via using thermal, electrical, or optical stimulus, and every state is long-term stable without any power supply [27]. So, the PCM has been extensively used in the nonvolatile memory [28], optical switch [29], optical modulation [30], optical computing [31], and optical neural network [32]. We should note, however, that these widely used PCMs have a common serious problem for the optical applications, that is the material absorption loss is very large in the optical communication bands. To address this issue, a new PCM Sb_2_Se_3_ is developed recently which has quite low optical loss (imaginary part < 10^−5^) in the optical communication bands even at the high-loss crystalline state [33,34], and other features are similar with the commonly used PCMs. Therefore, the new material Sb_2_Se_3_ might be very promising for the development of new and high-performance photonic devices.

In this paper, we propose a compact on-chip reconfigurable silicon waveguide mode conversion scheme. The key mode conversion section is formed by embedding a taper made of the material Sb_2_Se_3_ into the silicon waveguide. The taper is embedded asymmetrically on one side relative to the centerline of the silicon waveguide to convert the input TE_0_ mode to TE_1_ mode at the output. We add graphene and alumina (Al_2_O_3_) layers on the top surface of the material Sb_2_Se_3_ to act as a microheater, which is required to achieve the phase transition of the material Sb_2_Se_3_. When the material Sb_2_Se_3_ operates at the crystalline state, its refractive index increases and is larger than that of silicon. The embedded asymmetrical tapering structure performs the TE_0_-to-TE_1_ mode conversion. When the material Sb_2_Se_3_ works at the amorphous state, its refractive index is very close to that of silicon. The embedded tapering structure has little impact on the mode transmission, resulting in TE_0_ mode at the output. Therefore, reconfigurable mode conversions between TE_0_ and TE_1_ mode can be achieved by controlling the operating state of the material Sb_2_Se_3_, while previous reports nearly do not have such reconfigurable function [15,16,17,18,19,20,21,24,25,26]. Our calculations show that the required mode conversion length is only 2.3 μm, which is quite shorter than most previous reports [15,16,17,19,20,21,24,25]. The mode conversion efficiency (CE), mode crosstalk (CT), and insertion loss (IL) are 97.5%, −20.5 dB, and 0.2 dB, respectively, at wavelength λ = 1550 nm for the TE_0_-to-TE_1_ mode conversion, where the achieved low IL is benefited from the quite low optical loss of material Sb_2_Se_3_ compared with other PCMs [33,34]. The performance for the reconfigurable function, i.e., TE_0_-to-TE_0_ mode, is better than that of TE_0_-to-TE_1_ mode conversion. Moreover, we can achieve reconfigurable TE_0_-to-TE_2_ mode conversion by changing the embedded tapering structures. Other reconfigurable mode conversions (TE_0_-to-TE_n_, n ≥ 3) can also be obtained in theory. Therefore, with the obvious advantages of reconfigurability, small size, high performance, and functional extensibility, the proposed mode conversion scheme can form the building blocks for on-chip multimode photonics in future reconfigurable and programmable PICs [35,36,37].

## 2. Device Structure and Principle

### 2.1. The Device Structure Design and Materials

**Figure 1:**
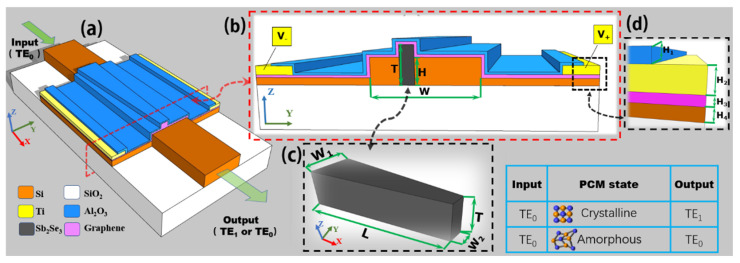
(**a**) Three-dimensional schematic of the proposed compact reconfigurable silicon waveguide mode converter. (**b**) Cross-sectional view of the mode conversion region of the proposed device. (**c**) Side view of the embedded PCM layer. (**d**) Enlarged view of the metal contacting components including the different material layers. The table shows the main device function. For input TE_0_ mode, the output can be switched between either TE_1_ or TE_0_ mode depending on the state of the PCM. The material and structural parameters of the proposed device are as labelled in the figures.

Figure 1 shows the schematic of the proposed reconfigurable silicon waveguide mode converter. The insets show the enlarged cross-sectional view of the mode conversion region and side view of the embedded PCM layer. To create an efficient refractive index change on the silicon waveguide, we embedded a PCM taper into the silicon waveguide by using waveguide etching and magnetron sputtering [38,39], where the end widths and length of the PCM taper are *W*_1_, *W*_2_, and *L*, respectively, and the PCM thickness is *T*. The position of the embedded PCM taper should be located asymmetrically to one side relative to the centerline of the silicon waveguide, to allow accumulation of the required π phase difference for the TE_0_-to-TE_1_ mode conversion [18,19]. For the PCM, here we use a new Sb_2_Se_3_ material rather than the conventionally used VO_2_, Ge_2_Sb_2_Te_5_, and Ge_2_Sb_2_Se_4_Te_1_ [33,34,40,41,42]. The reason is that Sb_2_Se_3_ has quite low optical loss compared with other PCMs. Furthermore, the phase transition temperature and melting temperature of Sb_2_Se_3_ are lower than that of silicon, thus the silicon waveguide will not be damaged during the phase transition process. The material Sb_2_Se_3_ is nonvolatile which means the proposed device will not require static power consumption, because the phase state (crystalline or amorphous state) of the material Sb_2_Se_3_ can sustain a long time without any power being supplied. Furthermore, Sb_2_Se_3_ can be switched between the crystalline and amorphous state over 4000 cycles without obvious aging symptoms [33,34]. Therefore, the material Sb_2_Se_3_ enables the proposed reconfigurable mode converter to work at low loss and low power consumption.

Further, we add graphene and Al_2_O_3_ layers atop the Sb_2_Se_3_. The thicknesses of the Al_2_O_3_ and graphene layers are *H*_1_ (=20 nm) and *H*_3_ (=0.35 nm), respectively. The graphene layer works as an efficient microheater because of its high thermal conductivity and low heat capacity [43,44]. The optical absorption loss of graphene could be quite low as the chemical potential of graphene is larger than 0.4 eV owing to the Pauli blocking mechanism [45,46]. The Al_2_O_3_ layer is used to prevent the graphene from oxidation. The thickness of the metal electrodes on both sides of the proposed device is chosen as *H*_2_ (=100 nm), and we also introduce a silicon slab layer with a thickness of *H*_4_ (=50 nm) to facilitate rapid heat dissipation when the Sb_2_Se_3_ layer changes from the crystalline state to amorphous state. The width and thickness of the input and output silicon waveguide are *W* (=1.1 μm) and *H* (=220 nm), respectively.

### 2.2. The Device Working Principle and Calculation Method

**Figure 2:**
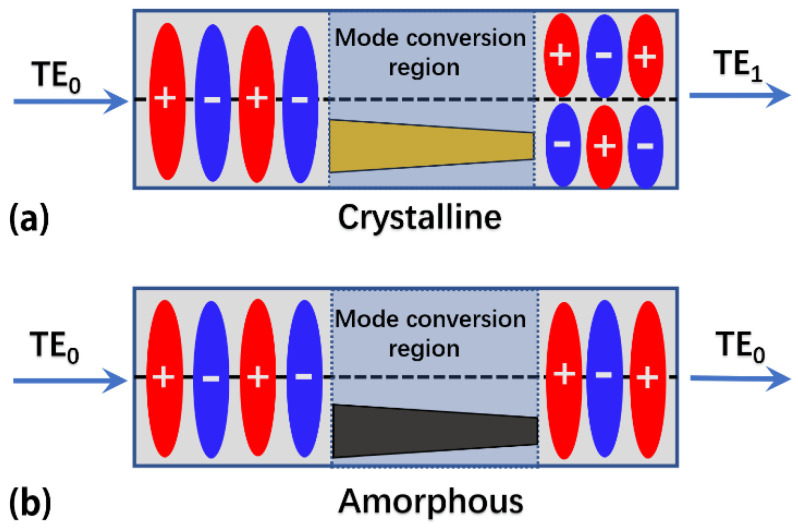
Schematic of mode conversion in the two phase states of the Sb_2_Se_3_ layer. (**a**) Mode conversion from input TE_0_ mode to output TE_1_ mode when the Sb_2_Se_3_ layer is in the crystalline state. (**b**) No mode conversion when the Sb_2_Se_3_ layer is in the amorphous state. Yellow (gray) color of Sb_2_Se_3_ layer represents the crystalline (amorphous) state. The symbols “+” and “−” represent the positive and negative phase of the electric field mode.

The device working principles are analyzed as follows. First when the Sb_2_Se_3_ layer is at the crystalline state, its optical refractive index is 4.05 at *λ* = 1.55 μm (imaginary part < 10^−5^) which is larger than that of silicon (~3.4) [33]. The asymmetrically placed Sb_2_Se_3_ layer relative to the centerline of the silicon waveguide creates two regions with different refractive index. One region is pure silicon with uniform refractive index distribution and the other region is silicon embedded with the Sb_2_Se_3_ layer with nonuniform refractive index distribution. The two regions are located on either side of the central axis along the waveguide transmission direction, as shown in Figure 2a. When the input TE_0_ mode enters the mode conversion region, it will be separated into two beams because of the different refractive indices at the end face of the mode conversion region. These two beams will then propagate along the two regions separately, i.e., the region with pure silicon waveguide and the other region which has a Sb_2_Se_3_ layer embedded in the silicon waveguide. Owing to different refractive index distribution between these two regions, the two transmitted beams will have different propagation constants and the phase difference between them will accumulate through the light propagation. When the accumulated phase difference between the beams in the two regions equals to π, we combine these two beams and connect to the output waveguide. The output is TE_1_ mode because the two beams have a π phase difference. The length of the mode conversion region is <3 μm and the conversion loss is <0.3 dB, because of the relatively large refractive index and low optical loss of Sb_2_Se_3_ material at the crystalline state.

When the Sb_2_Se_3_ layer switches from crystalline sate to amorphous state, its optical refractive index is 3.28 at *λ* = 1.55 μm (the imaginary part negligible) which is close to the refractive index of silicon (~3.4) [33]. Such a small refractive index difference in a short propagation length (<3 μm) has negligible effect on the mode field transmission. As a result, the input TE_0_ mode will be transmitted through the mode conversion region without any mode conversion, as shown in Figure 2b. In summary, by switching between the phase states of the embedded Sb_2_Se_3_ layer, one can obtain either TE_1_ or TE_0_ mode at the device output for the same input TE_0_ mode, thus achieving reconfigurable mode conversion.

To analyze the device performance and optimize the device parameters, three-dimensional finite-difference time-domain (3D-FDTD) method was employed [47,48], which could well calculate the mode transmission and conversion performance of the proposed device. In the following sections, we will use the 3D-FDTD method to find the optimum structural parameters based on the above-mentioned device working principle.

## 3. Results and Discussion

Before we conduct the calculation and optimization of the device performance, the key device performance indictors should be defined at first. Here, we use the device performance indicators of mode CE, CT, and IL to characterize the device performance. For the TE_0_-to-TE_1_ mode conversion, the mode CE is defined as [19,20]

(1) $$\mathrm{CE}=\frac{P_{{\mathrm{TE}}_{1}}}{P_{\mathrm{out}}}\times 100\%,$$

where *P*_TE_1__ and *P*_out_ stand for the receiving power of TE_1_ mode and total output power at the device output port, respectively. Mode CT is defined as [19,20]

(2) $$\mathrm{CT}=\max\left({10\log}_{10}\frac{P_{\mathrm{OT}}}{P_{\mathrm{TE}}{}_{{}_{1}}}\right),$$

where *P*_OT_ represents the output power of the other interfering mode rather than TE_1_ at the device output port and we choose the maximum value as the mode CT for the proposed device. IL is defined as [19,20]

(3) $$\mathrm{IL}=-\log_{10}\frac{P_{{\mathrm{TE}}_{1}}}{P_{\mathrm{in}}},$$

where *P*_in_ represents the power of the input TE_0_ mode at the device input port. If not otherwise specified, the working wavelength is set as 1.55 μm in the following discussion. We then carried out extensive numerical simulation to determine the structural parameters of the embedded Sb_2_Se_3_ layer for optimal device performance. The optimized values of embedded Sb_2_Se_3_ layer are as follows: the embedded taper end widths *W*_1_ = 340 nm and *W*_2_ = 100 nm, the layer thickness *T* = 340 nm, the lateral shift relative to the waveguide center *S* = 360 nm, and the taper length *L* = 2.3 μm.

**Figure 3:**
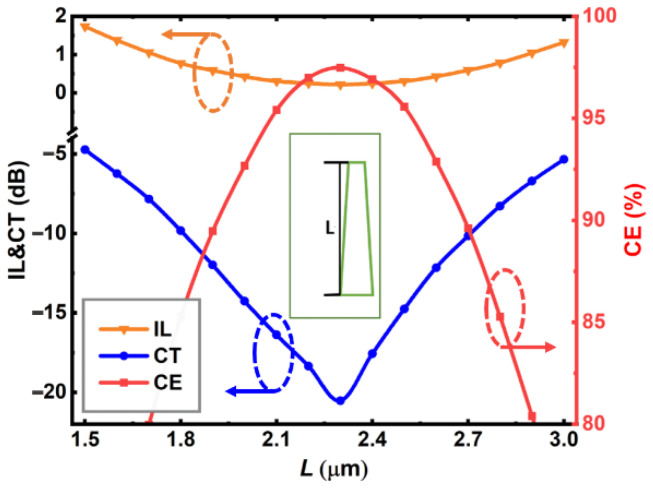
Calculated mode CE, CT, and IL of the proposed mode converter as a function of the PCM (Sb_2_Se_3_) taper length *L*. The embedded Sb_2_Se_3_ layer works at the crystalline state and the inset shows the calculated taper length.

Figure 3 shows the calculated mode CE, CT, and IL of the proposed device as a function of the PCM (Sb_2_Se_3_) taper length *L*, where the end widths and thickness of the Sb_2_Se_3_ layer are chosen at the respective optimal values *W*_1_ = 340 nm, *W*_2_ = 100 nm and *T* = 340 nm. For the TE_0_-to-TE_1_ mode conversion, the Sb_2_Se_3_ layer is in the crystalline state. From Figure 3, the mode conversion performance is closely related to the PCM taper length within the calculation range from *L* = 1.5 to 3.0 μm. The optimum performance is obtained at *L* = 2.3 μm with the highest CE = 97.5%, lowest CT = −20.5 dB, and lowest IL = 0.2 dB. For a fabrication error of ±0.2 μm, i.e., *L* varies from 2.1 to 2.5 μm, the device performance is still acceptable with CE > 95%, CT < −15 dB, and IL < 0.3 dB. The taper length *L* is chosen at 2.3 μm in the following discussion, where such conversion length is clearly shorter than most previous reports [15,16,17,19,20,21,24,25].

**Figure 4:**
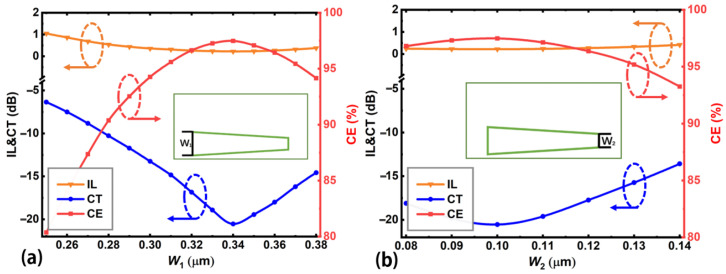
Device performance (mode CE, CT, and IL) as functions of the (**a**) input end width *W*_1_ and (**b**) output end width *W*_2_ of the embedded Sb_2_Se_3_ layer for the proposed mode converter. Insets show the definition of the device parameters being calculated.

Figure 4 shows the effect of the end widths (*W*_1_, *W*_2_) of the embedded Sb_2_Se_3_ layer on the device performance. For *W*_1_, the Sb_2_Se_3_ layer will reach the waveguide boundary if *W*_1_ is larger than 380 nm. So, we set *W*_1_ ≤ 380 nm in the calculations. For *W*_2_, the achievable width depends on the state of fabrication technology. We choose *W*_2_ ≥ 80 nm, which could be achieved using current E-beam lithography and etching processes [49,50]. The Sb_2_Se_3_ layer thickness *T* and lateral shift *S* are set at the optimal values of 340 nm and 360 nm, respectively. Figure 4 shows that the input end width *W*_1_ has stronger effect on the device performance when compared with the output end width *W*_2_. The widths for the best device performance are at *W*_1_ = 340 nm and *W*_2_ = 100 nm, corresponding to CE = 97.5%, CT = −20.5 dB, and IL = 0.2 dB. Assuming a device performance of CE > 95%, CT < −15 dB, IL < 0.3 dB, *W*_1_ and *W*_2_ should be within the ranges of [310, 370] nm and [80, 130] nm, respectively. More detailed comparisons of the mode conversion performance (CE, CT, IL) with other reports can be found in the following Table 1.

**Figure 5:**
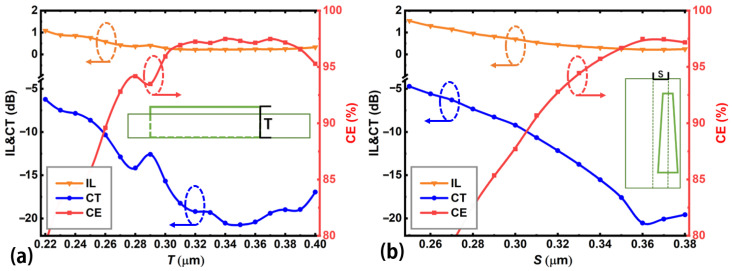
Device performance (mode CE, CT, and IL) as functions of the (**a**) Sb_2_Se_3_ layer thickness *T* and (**b**) lateral shift *S* of the embedded Sb_2_Se_3_ layer relative to the centerline of the waveguide. Insets show the definition of the device parameters being calculated.

Figure 5 plots the device performance versus the Sb_2_Se_3_ layer thickness *T* and lateral shift *S* of the embedded Sb_2_Se_3_ layer relative to the waveguide center. The taper widths *W*_1_ and *W*_2_ are set as 340 nm and 100 nm, respectively. The insets of Figure 5a,b show the definition of *T* and *S*, respectively. From Figure 5a, we find that the device performance would be very poor if the thickness of the Sb_2_Se_3_ layer *T* is the same as that of the silicon waveguide (*H* = 220 nm). So, it is necessary to choose different thicknesses which would require extra fabrication steps during device fabrication. The optimum thickness of Sb_2_Se_3_ layer is 340 nm, corresponding to the performance CE = 97.5%, CT = −20.5 dB, and IL = 0.2 dB. The inset in Figure 5a shows the definition of the Sb_2_Se_3_ layer thickness *T*. For the same device performance criteria mentioned above, i.e., CE > 95%, CT < −15 dB, IL < 0.3 dB, the thickness *T* can vary from 300 to 400 nm. The large tolerance in thickness *T* relaxes the constrains on device fabrication. Figure 5b shows that the lateral shift *S* of the embedded Sb_2_Se_3_ layer relative to the centerline of the waveguide has a strong effect on the device performance when compared with other parameters. The reason is that the lateral shift *S* determines the refractive index distribution in the mode conversion region. When the lateral shift changes, the corresponding refractive index distribution will change, strongly affecting the mode conversion performance [19,20]. The optimum lateral shift is found to be 360 nm relative to the centerline of the waveguide. Note that the embedded Sb_2_Se_3_ layer will be outside the silicon waveguide boundary for *S* > 380 nm. Thus, the calculation range of *S* should be less than 380 nm. In summary, the input end width *W*_1_ and lateral shift *S* should be carefully controlled during the fabrication process since their fabrication tolerances are relatively small.

**Figure 6:**
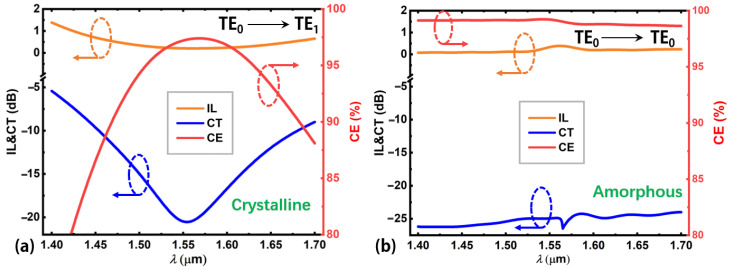
The mode CE, CT, and IL versus wavelength of the proposed mode converter working at the (**a**) crystalline sate and (**b**) amorphous state. The wavelength range is from 1.4 to 1.7 μm.

Figure 6a depicts mode CE, CT, and IL as a function of wavelength for the proposed TE_0_-to-TE_1_ mode converter, when material dispersions are considered [33,51]. Figure 6b shows the reconfigurable function, i.e., no mode conversion when the embedded Sb_2_Se_3_ layer is at the amorphous state. Figure 6a shows a strong wavelength dependence in the range from 1.4 to 1.7 μm for the mode conversion from TE_0_ to TE_1_ mode. By contrast, Figure 6b shows that when no mode conversion takes place, the device performance exhibits only a small wavelength dependence. Again, for the same device performance criteria (CE > 95%, CT < −15 dB, IL < 0.3 dB), the allowable working wavelength range is from 1507 nm to 1616 nm (bandwidth = 109 nm) for the TE_0_-to-TE_1_ mode conversion. As for the reconfigurable function, the corresponding CE, CT, and IL are >98.5%, <−24 dB, and <0.23 dB, respectively, in the wavelength range from 1.4 to 1.7 μm. Thus, the proposed mode converter has a good reconfigurable function, and the allowable working bandwidth covers the main optical communication bands, which is larger than the working bandwidths of some reported mode converters [16,18,24,25].

The main process to fabricate the proposed reconfigurable mode converter can be divided into three sections: fabricating the silicon waveguide, embedding the Sb_2_Se_3_ layer into the silicon waveguide, and depositing the graphene and Al_2_O_3_ layers atop the device including the metal contacts. We can start from a standard SOI wafer with a top silicon layer thickness of 220 nm and a buried oxide layer thickness of 2 μm. First, the silicon waveguide with a width of 1.1 μm and a slab layer thickness of 50 nm, including a taper slot, is fabricated on the SOI wafer using E-beam lithography and reactive ion etching processes [49,50]. Second, a Sb_2_Se_3_ layer with a thickness of 340 nm is deposited on the mode conversion region using magnetron sputtering [33,34], and then the Sb_2_Se_3_ material is removed except in the taper slot region such that the Sb_2_Se_3_ material will fill the etched slot region. More details about the film preparation and deposition of the Sb_2_Se_3_ material can refer to the work reported in [52]. Third, a graphene layer grown by the chemical vapor deposition is transferred onto the device surface. The metal contacts are added on both sides of the conversion region. Then, using atom layer deposition, a 20-nm-thick Al_2_O_3_ layer is deposited atop the graphene layer to prevent the graphene from oxidation [45]. Using these methods, we can realize the proposed reconfigurable silicon waveguide mode converter. Within these fabrication processes, if a small number of voids are introduced into the Sb_2_Se_3_ layer due to the sputtering error, the IL of the device might be slightly increased, but the mode CE and CT could be still guaranteed through further structural optimizations.

**Figure 7:**
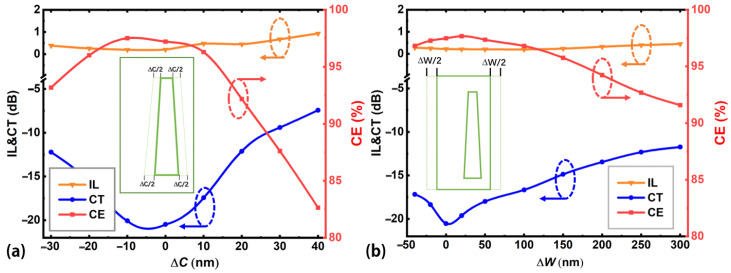
Fabrication tolerance analyses. Device performance as a function of the variation in the size of the (**a**) embedded Sb_2_Se_3_ layer Δ*C* and (**b**) silicon waveguide Δ*W* along the width direction. Insets in Figure 7a,b show the definition of Δ*C* and Δ*W* in the calculated waveguide structures, respectively.

**Figure 8:**
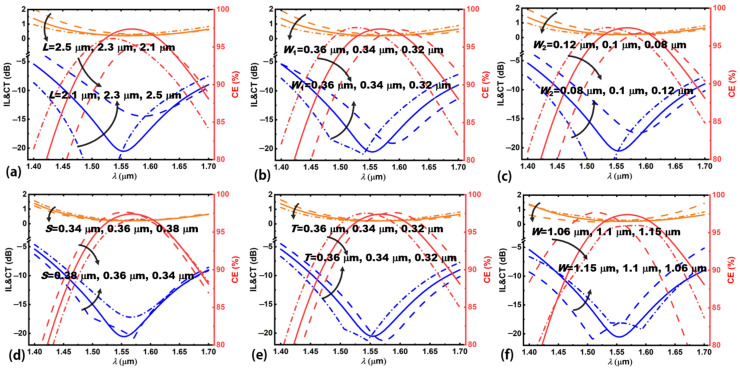
Wavelength spectra of mode CE, CT, and IL of the proposed device as the following parameters change, (**a**) taper length *L*, (**b**) input end width *W*_1_, (**c**) output end width *W*_2_, (**d**) lateral shift *S* relative to the centerline of the waveguide, (**e**) Sb_2_Se_3_ layer thickness *T*, and (**f**) silicon waveguide with *W*. The wavelength range is calculated from 1.4 to 1.7 μm.

To study the deterioration of the device performance caused by fabrication errors in practice, we analyze the effect of the variations of the size of the embedded Sb_2_Se_3_ layer (Δ*C*) and silicon waveguide (Δ*W*) along the width direction (*y*-direction in Figure 1b) on the device performance. Figure 7 shows the device performance deteriorates as Δ*C* or Δ*W* deviates from their optimum values. Insets in Figure 7a,b show the definition of Δ*C* and Δ*W*, respectively. For the same device performance criteria of CE > 95%, CT < −15 dB, and IL < 0.3 dB, Δ*C* and Δ*W* should be controlled within the ranges of −22 to 14 nm, and −50 to 150 nm, respectively. These tolerance requirements can be achieved using current fabrication facilities [49,50]. Figure 8 shows the wavelength spectra of the proposed device when the structural parameters (*L*, *W*_1_, *W*_2_, *S*, *T*, *W*) change. For the analysis of every structural parameter, other structural parameters are fixed at their optimal values. From Figure 8, the optimum working wavelength will shift as these calculated structural parameters vary from their optimal values, and the corresponding device performance will also deteriorate. For optimum device performance, one should target the determined structural parameters when fabricating the device.

**Figure 9:**
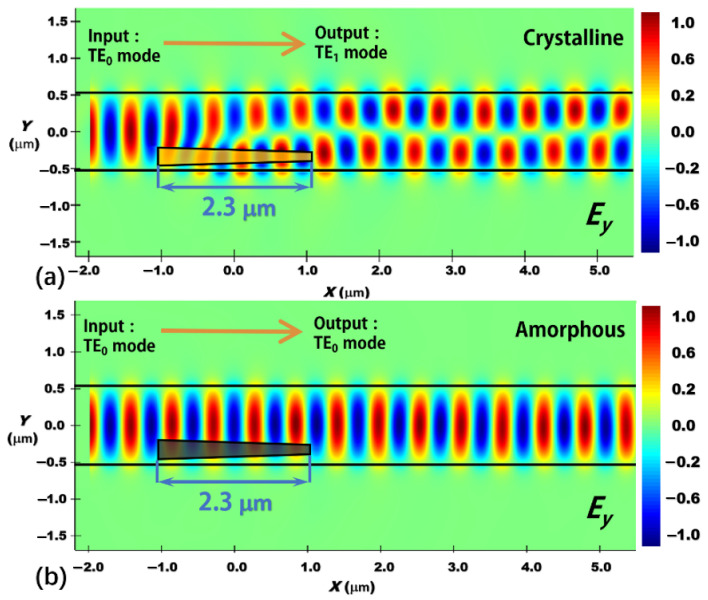
Evolutions of the electric field (dominant component: *E*_y_) along the propagation direction (*x*-direction) through the proposed mode converter. (**a**) Sb_2_Se_3_ layer working at the crystalline state and (**b**) Sb_2_Se_3_ layer working at the amorphous state. The mode conversion length is 2.3 μm and the working wavelength is 1.55 μm.

Figure 9 plots the evolution of the electric field along the propagation direction through the proposed reconfigurable mode converter. The mode conversion length is 2.3 μm and the 3D-FDTD method is used to perform the calculations. From Figure 9, either the TE_1_ or TE_0_ mode can be obtained at the device output port by switching the phase state of the embedded Sb_2_Se_3_ layer in the device. Because of the nonvolatile property of the Sb_2_Se_3_ material, either output (TE_1_ or TE_0_ mode) of the proposed device can be kept for a long time without consuming any energy, i.e., zero static power consumption [33,34,38,39]. When the Sb_2_Se_3_ layer is at the crystalline state, the input TE_0_ mode will be split into two beams when it enters the mode conversion region. The two beams transmit along the two channels. One channel is a pure silicon waveguide and the other channel is a silicon waveguide embedded with a Sb_2_Se_3_ layer. Because of the different mode propagation constants in these two channels, the split modes will accumulate phase difference between them during mode propagation. When the phase difference equals to π, the two beams are in opposite phase resulting in the TE_1_ mode, as shown in Figure 9a. When the Sb_2_Se_3_ layer is at the amorphous state, no mode conversion is observed and the input TE_0_ mode propagates through the device with little distortion as shown in Figure 9b. We obtain the TE_0_ mode at the output port of the device. Figure 9 shows that reconfigurable mode conversion can be achieved within a device length of only 2.3 μm. To the best of our knowledge, such reconfigurable function and conversion length have not been realized before [15,16,17,18,19,20,21,24,25,26].

**Figure 10:**
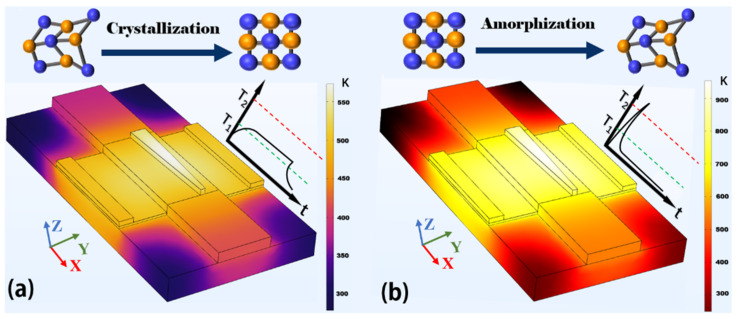
Electric-thermal simulation for the proposed reconfigurable mode converter. The phase state of Sb_2_Se_3_ material changes (**a**) from the amorphous state to the crystalline state and (**b**) from the crystalline state to the amorphous state, respectively. *T*_1_ and *T*_2_ stand for the phase transition temperature and melting temperature of Sb_2_Se_3_, respectively. The upper temperature limit of the proposed device is 1100 K.

Next, we analyze the phase change process between the crystalline state and amorphous state of the embedded Sb_2_Se_3_ layer. The electric-thermal phase transition method is employed based on the graphene micro-heater and the electric-thermal simulation is carried out using COMSOL Multiphysics [53]. Figure 10 shows the results of the electric-thermal simulation when the Sb_2_Se_3_ material undergoes the crystallization and amorphization process. The phase transition temperature of Sb_2_Se_3_ is 473 K and the melting temperature of Sb_2_Se_3_ is 893 K [33,34], where the upper temperature limit of the proposed device is 1100 K. From Figure 10, the required temperatures for the phase change of Sb_2_Se_3_ material can be achieved using the proposed graphene microheater. The graphene microheater is more efficient than other metal heaters because of the high thermal conductivity and low heat capacity of graphene [43,44]. In addition, the phase transition and melting temperatures of Sb_2_Se_3_ are clearly lower than the melting temperatures of silicon, silica, graphene, and Al_2_O_3_, thus the phase transition process will not damage the proposed device. For the switching time of the material Sb_2_Se_3_ based on the electric-thermal phase transition method, the commonly required pulse width is ~100 μs (120 μs for the trailing edge) from the amorphous state to crystalline state, while the pulse width is only ~400 ns (10 ns for the trailing edge) from the crystalline state to the amorphous state [44]. So, one switching cycle is <250 μs, including the trailing edge of the pulse. So, the proposed mode converter can therefore utilize the phase change property of Sb_2_Se_3_ to perform the reconfigurable mode conversion functions.

**Figure 11:**
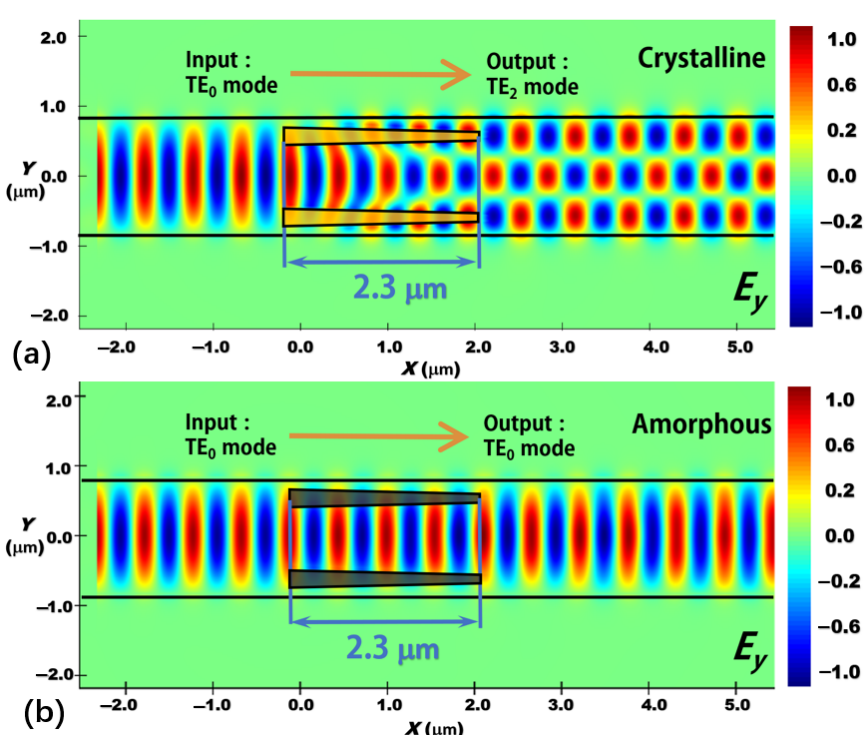
Evolutions of the electric field (dominant component: *E*_y_) along the propagation direction (*x*-direction) through a reconfigurable TE_0_-to-TE_2_ mode converter. (**a**) The Sb_2_Se_3_ layer working at the crystalline state and (**b**) the Sb_2_Se_3_ layer working at the amorphous state. The mode conversion length is still 2.3 μm and the working wavelength is 1.55 μm.

**Figure 12:**
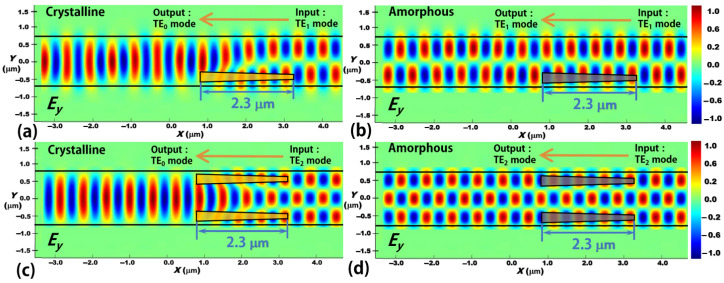
Backward transmission processes of the proposed reconfigurable mode converters. Evolutions of the electric field (dominant component: *E*_y_) along the propagation direction (*x*-direction) through the reconfigurable TE_1_-to-TE_0_ mode converter, when the Sb_2_Se_3_ layer working at (**a**) the crystalline state and (**b**) the amorphous state, respectively. Evolutions of the electric field (dominant component: *E*_y_) along the propagation direction (*x*-direction) through the reconfigurable TE_2_-to-TE_0_ mode converter, when the Sb_2_Se_3_ layer working at (**c**) the crystalline state and (**d**) the amorphous state, respectively.

We then apply the principle of the proposed reconfigurable mode conversion scheme for TE_0_-to-TE_1_ to realize reconfigurable higher-order mode converters. We design a reconfigurable TE_0_-to-TE_2_ mode converter, in which two Sb_2_Se_3_ tapers are embedded into the silicon waveguide symmetrically with respect to the centerline of the silicon waveguide, as shown in Figure 11. The mode conversion length is still 2.3 μm. From the simulation results, the conversion performance from TE_0_ to TE_2_ mode is quite good with CE = 99.4%, CT < −25.2 dB, and IL = 0.11 dB at *λ* = 1.55 μm. When the Sb_2_Se_3_ layer switches from crystalline to amorphous state, the reconfigurable function can be obtained with no mode conversion. Thus, the reconfigurable function of the TE_0_-to-TE_2_ mode converter is achieved. Moreover, we also study the backward transmission processes of the proposed TE_0_-to-TE_1_ and TE_0_-to-TE_2_ reconfigurable mode converters, where the higher-order modes (TE_1_ mode and TE_2_ mode) are injected from the right port under two types of the phase state conditions, as illustrated in Figure 12. From Figure 12, we can clearly find that both TE_1_ and TE_2_ modes can be well converted to the fundamental TE_0_ mode when the Sb_2_Se_3_ layer works at the crystalline state. Meanwhile, no mode conversion can happen when the Sb_2_Se_3_ layer works at the amorphous state and the output modes are still TE_1_ mode and TE_2_ mode, respectively. In principle, other reconfigurable higher-order mode converters can also be designed by using our previously reported extension rule [54]. For the reconfigurable function of the present device, it is just like a mode switch, which can be switched between the output TE_0_ mode and TE_1_ (or TE_2_) mode. While some previously reported mode converters normally have only one function for a device [15,16,17,18,19,20,21,24,25,26] with quite low functional flexibility. When these devices are designed and fabricated, their functions are determined which cannot be further changed. If we program signals on these two outputting modes based on the proposed reconfigurable mode converter and combine with other components on the same chip, the whole chip could have a programmable function. Further, if we add more reconfigurable mode converters in the PIC, more functions will be obtained for the same PIC, which could support more applications (e.g., optical computing [31], optical neural network [32], optical imaging [55]).

Table 1 compares the proposed mode converters with typical mode converters reported recently in the literature. We consider the device structure, function, size, performance, and reconfigurability. From Table 1, the proposed devices have obvious advantages in conversion length, device performance, and reconfigurable functions. By comparison, we can also easily find the superiority of the proposed reconfigurable mode converters, which could well support the development of on-chip multimode photonics.

Finally, with the features of short conversion length (2.3 μm), high conversion performance (CE > 97%, CT < −20 dB, IL~0.2 dB), reconfigurable mode conversion, and functional extensibility, we believe the proposed reconfigurable silicon waveguide mode conversion scheme and related devices could find important applications in on-chip multimode photonics and be one of the fundamental building blocks for the reconfigurable multimode PICs [13,14].

## 4. Conclusions

In conclusion, we proposed a reconfigurable silicon waveguide mode conversion scheme, in which the reconfigurable function is achieved by using nonvolatile and low-loss optical phase change material Sb_2_Se_3_. A hybrid Sb_2_Se_3_-silicon waveguide is obtained by embedding a tapered Sb_2_Se_3_ layer into the silicon waveguide. To achieve the mode conversion from input TE_0_ to output TE_1_ mode, the embedded Sb_2_Se_3_ layer should be located on one side of the centerline of the silicon waveguide. When the Sb_2_Se_3_ layer works at the crystalline state, the input TE_0_ mode can be efficiently converted to TE_1_ mode at the output in a device length of only 2.3 μm, which is much shorter than most reports. When the Sb_2_Se_3_ layer works at the amorphous state, no mode conversion occurs, which corresponds to the reconfigurable mode conversion. Note that the reconfigurable function cannot be achieved using previous mode converters. From the simulation results, the device performance for the TE_0_-to-TE_1_ mode conversion is CE = 97.5%, CT < −20.5 dB, and IL = 0.2 dB, where the quite low IL is benefited from the low-loss feature of the employed material Sb_2_Se_3_. We also analyze the device working bandwidth and fabrication tolerance of key structural parameters, as well as carry out the electric-thermal simulation for the phase change process. In addition, the present device scheme can be extended to realize other reconfigurable higher-order mode conversions (e.g., TE_0_-to-TE_2_ mode conversion, CE = 99.4%, CT < −25.2 dB, and IL = 0.11 dB at *λ* = 1.55 μm), demonstrating the extensibility of the proposed scheme. With these advantages, the proposed device scheme can provide reconfigurable higher-order mode sources for on-chip multimode photonics.

## Figures and Tables

**Table 1:** Comparison of the proposed mode converter with typical mode converters reported in the literature.

Structure	Function	Length (μm)	CE (%)	CT (dB)	IL (dB)	BW (nm)	Reconfigurability
ADC [16]	TE_0_-to-TM_0_ [E]	44	>92	<−15	<1	40 (CE > 92%)	NO
DES [18]	TE_0_-to-TE_1_ [E] TE_0_-to-TE_2_ [E]	2.3 2.4	- -	~−10 ~−14	<0.5 <0.3	60 (CT < −7 dB) 50 (CT < −9 dB)	NO NO
SES [20]	TM_0_-to-TM_2_ [S]	6	~94	<−15	~0.5	128 (CE > 94%)	NO
TMS [24]	TE_0_-to-TM_1_ [S]	11	-	<−25	4.2	100 (CT < −20 dB)	NO
HPS [25]	TE_0_-to-TM_1_ [S]	7	94.6	-	<2.34	35 (CE > 92.2%)	NO
This work	TE_0_-to-TE_1_ [S]	2.3	97.5	<−20.5	0.2	210 (CE > 90%)	YES
	TE_0_-to-TE_2_ [S]	2.3	99.4	<−25.2	0.11	245 (CE > 90%)	YES

DES: Deeply etched slot; SES: Shallowly etched slot; TMS: Tapered metal structure; HPS: Hybrid plasmonic slot; BW: Bandwidth; E: Experiment; S: Simulation; “-”: not mentioned.

## Data Availability

The data that support the findings of this study are available from the corresponding author upon reasonable request.
